# Poly(lactic acid)/Poly(3-hydroxybutyrate) Biocomposites with Differently Treated Cellulose Fibers

**DOI:** 10.3390/molecules27082390

**Published:** 2022-04-07

**Authors:** Adriana Nicoleta Frone, Marius Ghiurea, Cristian Andi Nicolae, Augusta Raluca Gabor, Stefania Badila, Denis Mihaela Panaitescu

**Affiliations:** National Institute for Research & Development in Chemistry and Petrochemistry–ICECHIM, 202 Splaiul Independentei, 060021 Bucharest, Romania; adriana.frone@icechim.ro (A.N.F.); ghiurea@gmail.com (M.G.); cristian.nicolae@icechim-pd.ro (C.A.N.); raluca.gabor@icechim.ro (A.R.G.); stefania.badila@yahoo.com (S.B.)

**Keywords:** biocomposite, PLA/PHB blend, cellulose fibers, high-temperature XRD

## Abstract

The growing concern about environmental pollution has generated an increased demand for biobased and biodegradable materials intended particularly for the packaging sector. Thus, this study focuses on the effect of two different cellulosic reinforcements and plasticized poly(3-hydroxybutyrate) (PHB) on the properties of poly(lactic acid) (PLA). The cellulose fibers containing lignin (CFw) were isolated from wood waste by mechanical treatment, while the ones without lignin (CF) were obtained from pure cellulose by acid hydrolysis. The biocomposites were prepared by means of a melt compounding-masterbatch technique for the better dispersion of additives. The effect of the presence or absence of lignin and of the size of the cellulosic fibers on the properties of PLA and PLA/PHB was emphasized by using in situ X-ray diffraction, polarized optical microscopy, atomic force microscopy, and mechanical and thermal analyses. An improvement of the mechanical properties of PLA and PLA/PHB was achieved in the presence of CF fibers due to their smaller size, while CFw fibers promoted an increased thermal stability of PLA/PHB, owing to the presence of lignin. The overall thermal and mechanical results show the great potential of using cheap cellulose fibers from wood waste to obtain PLA/PHB-based materials for packaging applications as an alternative to using fossil based materials. In addition, in situ X-ray diffraction analysis over a large temperature range has proven to be a useful technique to better understand changes in the crystal structure of complex biomaterials.

## 1. Introduction

The packaging industry is among the largest consumers of plastics and the greatest source of plastic waste, given the single use of packaging or its short lifespan [1]. This fact has indicated a major research interest for the design and development of biodegradable materials as substitutes for fossil-derived polymers in the packaging sector. Biodegradable and bio-based polymers from the family of aliphatic polyesters, such as poly(lactic acid) (PLA) or poly(3-hydroxybutyrate) (PHB), have received the greatest attention [2,3,4,5,6,7]. These show good properties and a great ability to be processed into sheets and complex forms, which is important for packaging [7]. Nevertheless, some thermal and mechanical properties of PLA and PHB are weaker than that of fossil-based polymers [3,4,5].

Many attempts have been made to improve these properties [4,5,8]. The most effective is the addition of cellulose reinforcements for enhancing the mechanical properties and plasticizers for a better processability. Cellulose nanofillers are primarily used to modify the properties of biopolymers due to their high stiffness and surface area, in addition to their availability and biodegradability [8]. In particular, cellulose nanocrystals (CNC) have been added to PLA using a melt mixing technique to enhance its crystallinity and stiffness, along with its biodegradability, while still maintaining transparency [9,10]. The surface treatment of CNC with 3-aminopropyltriethoxysilane improved its dispersion in PLA and the mechanical properties of the biocomposite [10]. Bacterial cellulose (BC) nanofibers obtained from BC pellicles by mechanical treatment enhanced the properties of PLA due to their high aspect ratio [11]. Acylated microcrystalline cellulose (MCC) obtained from rice bran oil was added to PLA by solvent casting in chloroform [12], leading to a high reduction of water vapor permeability, better mechanical properties, and thermal stability, along with excellent UV light barrier properties. The beneficial effect of CNC on the oxygen barrier properties of the extrusion blow-molded PLA/CNC films was also highlighted [13]. The surface modification of CNC by surfactants (lauric arginate) [14], cis-9-octadecenylamine [15], poly(glycidyl methacrylate) [16], or acetylation [17] was also tested for improving compatibility, along with the barrier and mechanical properties of PLA/CNC nanocomposites. However, all these attempts have used the solution casting method in dichloromethane/chloroform as the manufacturing process, raising issues related to the environmental and industrial applicability.

Blending PLA with PHB has emerged as an effective method to enhance the thermal, mechanical, and barrier properties of PLA [2,3,4,5,6,7]. Early studies have shown that PLA and PHB are immiscible, regardless of their ratio in the blends, but they exhibit molecular interaction [18]. The PLA/PHB 75/25 blend obtained by melt-mixing exhibited the best tensile properties compared with pure PLA, PHB, and blends with other components’ ratios [18]. Similarly, PLA and poly(3-hydroxybutyrate-co-3-hydroxyvalerate) (PHBV) were found to be immiscible in melt-mixed PLA/PHBV blends, showing two different glass transition temperatures (*T_g_*); however, a mutual influence of PLA and PHBV in the blends was observed from the shift of the *T_g_* values to each other [19]. Better properties were obtained after the addition of CNC or other cellulose reinforcements in the PLA/PHB blends [2,3,4]. Thus, improvements in tensile strength and modulus, with 25% and 63% respectively, were reported after the addition of 1 wt% CNC in PLA/PHB 75/25 blend [20]. A higher storage modulus of extruded filaments and 3D printed parts was obtained after reactive blending using dicumyl peroxide as a cross-linking agent [3]. 

Ductile properties and an increased flexibility are required in most food packaging applications, which cannot be obtained with neat PLA/PHB blends or nanocomposites [21]. Therefore, the combined effect of PHB and acetyl(tributyl citrate) (ABC) or other plasticizers on PLA properties was intensively studied [2,7,21,22,23,24]. The PLA/PHB (75:25) blends plasticized with 15 wt% ABC showed good flexibility (over 80 times higher elongation at break compared to PLA) and transparency and reduced water absorption, along with enhanced oxygen barrier properties, which recommends them for biodegradable food packaging [21]. The PLA/PHB (70/30) blends plasticized with 15 wt% glyceryl tributyrate showed better fracture toughness and ductility compared to pure PLA or PHB, along with good water vapor permeation, with their properties being close to that of polyethylene films largely used in packaging [7,22,23]. The combined effect of ABC and CNC in PLA/PHB blends was also studied [2,24]. These plasticized nanocomposites showed a better dispersion of CNC, good transparency, improved oxygen barrier properties, more rapid disintegration under composting conditions, and increased flexibility [2,24]. However, all these blends and biocomposites showed a much lower modulus compared to that of neat PLA. 

The results published so far are focused only on CNC, surface modified or not, as a reinforcement in PLA/PHB composites [2,24,25]. The influence of lignin on the properties of these composites has not yet been studied. Previous works have shown the beneficial effect of lignin in PHB [26] or PLA [27] composites containing cellulose fibers. Thus, wood-waste cellulose fibers containing lignin improved the thermal stability, crystallinity, and mechanical properties of plasticized PHB, acting as an efficient and low-cost modifier [26]. Similarly, lignin enhanced the interfacial adhesion between PLA and cellulose fibers, leading to improved thermal, mechanical, and barrier properties [27,28]. Therefore, the influence of cellulose fibers containing lignin (CFw), isolated from wood waste by mechanical treatment, and cellulose fibers obtained from pure cellulose (CF) (without lignin) by acid hydrolysis, on the properties of a PLA/PHB blend with a low content of plasticizer has been studied in this work. The biocomposites were obtained by melt compounding using a masterbatch technique, which is environmentally benign and easily scalable. For the first time, in situ X-ray diffraction results from room to melting temperature were correlated with differential scanning calorimetry and polarized optical microscopy results to better understand the influence of cellulose fibers with or without lignin on the crystal structure of plasticized PLA/PHB biocomposites in different temperature environments. This is of utmost importance for the practical application of these materials. 

## 2. Materials and Methods

### 2.1. Materials

Polylactic acid (PLA) Ingeo 4043D, with a content of 98% L-lactide, an average molecular weight (M_w_) of 111 kDa, and a density of 1.24 g/cm^3^ was purchased from NatureWorks (Minnetonka, MN, USA). PHB powder with M_w_ 490 kDa and a density of 1.25 g/cm^3^ was purchased from Biomer (Schwalbach am Taunus, Germany) and acetyl tributyl citrate (ABC) plasticizer from Sigma-Aldrich (Burlington, MA, USA). Microcrystalline cellulose (MCC) powder with a mean particle size of 20 µm was supplied by Sigma-Aldrich (Schnelldorf, Germany). Mixed softwood and hardwood waste was collected from wood exploitations and timber factories. Sulfuric acid 96% from Fluka (Buchs, Switzerland) was used for the treatment of MCC.

### 2.2. Preparation of Cellulose Fibers

CF cellulose fibers were obtained from MCC, a pure cellulose source, by acid hydrolysis. Sulfuric acid was added, drop by drop, in a water suspension of MCC (1/10 wt%) up to a final concentration of 60%. The hydrolysis was conducted at 40 °C for 150 min. After hydrolysis, the suspension containing CF was first centrifugated and then dialyzed using regenerated cellulose membrane (Carl Roth Spectra/Por 1) until it reached a final pH of 6 and then frozen at −20 °C for 48 h. CF was freeze-dried using a FreeZone 2.5 L system (Labconco, Kansas City, MO, USA) at −84 °C under vacuum (0.008 mbar) for 72 h, resulting in a fine powder.

CFw cellulose fibers were obtained from soft and hard wood waste mixtures by the procedure described in [26], with several differences: after auto-hydrolysis using high-pressure saturated steam and defibrillation, the cellulose fibers were dried for 24 h at 50 °C and were passed three times through a ball mill for 8 h each time. CFw has the following characteristics: 21% Klason lignin (TAPPI T222 om, 1998 [29]), 50% cellulose (Kürschner and Hoffer method [30]), 1.2% extractives in alcohol-benzene (TAPPI T204 cm, 1997 [31]), and 0.8% ash (TAPPI T211 om, 1993 [32]). 

### 2.3. Preparation of Masterbatches and Biocomposites

Plasticized PHB (PHB-ABC) was obtained from PHB powder and ABC (10%) in a 50 cm^3^ mixing cell (Brabender, Duisburg, Germany) at 165 °C for 8 min with a rotor speed of 50 min^−1^. The sensor located in the mixing chamber’s wall showed a temperature between 170 and 172 °C in the melt. The same compounding conditions were used to obtain the masterbatches, one containing 5% CFw besides ABC and the other the same proportion of CF. Plasticized PHB and the two masterbatches were diluted with PLA using the same mixing cell and working at 170 °C, at 50 min^−1^ rotor speed for 8 min. The composition of the samples is presented in Table 1. A PLA/PHB ratio of 73/27 was chosen based on the best properties reported for these blends [3,23,24]. For the preparation of the films, the compounds were profiled on a Brabender rolling mill at 130 °C, followed by compression molding using a P200E press (Dr. Collin, Maitenbeth, Germany) in the following conditions: pre-heating at 175 °C for 150 s (0.5 MPa), pressing at the same temperature with 10 MPa for 60 s, and rapid cooling using a cooling cassette accessory. The films showed a uniform thickness of 0.080 ± 0.006 mm, measured in ten points with a micrometer with 0.001 mm accuracy. They were maintained at room temperature before characterization. The PLA reference was processed under the same conditions. 

### 2.4. Characterization

#### 2.4.1. Thermo-Gravimetric Analysis (TGA)

The thermal stability of PLA, ABC plasticized PHB, PLA/PHB blend, and biocomposites (PLA/PHB/CFw, PLA/PHB/CF) was investigated using a Q5000 TGA analyzer (TA Instruments Inc., New Castle, DE, USA) in the range between room temperature and 700 °C at a heating rate of 10 °C/min in a nitrogen atmosphere, with a flow rate of 40 mL/min.

#### 2.4.2. Differential Scanning Calorimetry (DSC)

The melting and crystallization behavior of the polymers, blend, and biocomposites was studied using a differential scanning calorimeter Q2000 (TA Instruments Inc., USA) under helium flow (100 mL/min). The samples were cooled to −60 °C, equilibrated for 2 min, heated to 215 °C (first heating), equilibrated for 2 min and cooled to −60 °C (cooling cycle), with both heating and cooling cycles using 10 °C/min. The crystallinity of PLA (*X_cPLA_*, %) was determined from the first heating scan using the following equation [20]:

(1) $$X_{cPLA}=\frac{\Delta H_{m}-\Delta H_{cc}}{\Delta H_{m}^{0}\times w}\times 100$$

Δ*H_m_* (J/g) and Δ*H_cc_* (J/g) are the melting and cold crystallization enthalpies, while $\Delta H_{m}^{0}$ is the melting enthalpy of 100% crystalline PLA (93 J/g [20]) and *w* (%) is the weight percentage of PLA in the samples. The crystallinity of PHB was calculated similarly, but considering that PHB has no cold crystallization event and the $\Delta H_{m}^{0}$ is 146 J/g [21].

#### 2.4.3. In Situ High-Temperature X-ray Diffraction (XRD)

The crystalline structure of the PLA and the biocomposites was analyzed by in situ X-ray diffraction in the temperature range of 30–170 °C under nitrogen flow using a SmartLab diffractometer (Rigaku Corporation, Japan) mounted in parallel beam configuration with a Cu X-ray rotating anode (λ = 0.1541 nm) operating at 45 kV, 200 mA. An Anton Paar CHC plus^+^ cryo & humidity chamber was used for the measurements. The heating rate was of 10 °C/min with a holding time of 5 min before each measurement. Data were collected over a 2θ range from 5° to 60° with 4°/min.

#### 2.4.4. Polarized Optical Microscopy (POM)

The surface morphology of PLA, PLA/PHB, and the biocomposite films was investigated with a polarized light microscope (Olympus BX53, Japan) in transmission mode, with the polarizer below the sample and the analyzer rotated 90° above. The films of about 25 µm in thickness were placed between two microscope glass covers and melted in an oven set to 220 °C for 5 min to eliminate any crystallization seeds, then isothermally crystallized at 130 °C for 24 h.

#### 2.4.5. Dynamic Mechanical Analysis (DMA)

A DMA Q800 system (TA Instrument Inc., USA) was used to measure the thermo-mechanical properties of the samples in the temperature range from −30 °C to 135 °C at a heating rate of 3 °C/min, in tension mode. The measurements were carried out at a frequency of 1 Hz on rectangular specimens of 13 mm × 7 mm × 0.08 mm (length × width × thickness), cut from the compressed plates. 

#### 2.4.6. Atomic Force Microscopy (AFM)

The morphology of the cellulose fibers, PLA/PHB, and the biocomposites films was investigated using a MultiMode 8 atomic force microscope from Bruker Nano Inc. (USA) equipped with a Nanoscope V controller and a NanoScope software version 1.20. The measurements were performed in Peak Force Quantitative Nanomechanical Mapping (PF-QNM) mode in air, using silicon nitride tips, at a constant scan rate of 1.0 Hz and a scan angle of 90°. The tips used for measurements had a cantilever length of 225 µm and a resonance frequency of 75 kH. Image processing and data analysis were performed using the NanoScope software.

## 3. Results and Discussion

### 3.1. Morphological investigation by AFM

**Figure 1:**
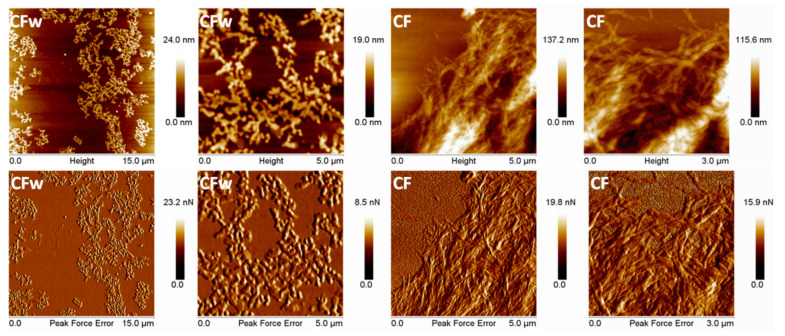
Topographic and PFE images with different magnification of CFw and CF.

AFM topographic and peak force error (PFE) images of CFw and CF are shown in Figure 1. The two types of fibers have different aspects and dimensions. Their average width calculated by the NanoScope 1.20 software is almost double in the case of CFw compared to CF, i.e., 70 ± 8 nm for CFw and 37 ± 8 nm for CF. Although the real widths of cellulose fibers are probably smaller than the measured ones, due to the AFM tip widening effect [33], the ratio between their widths is high. The length of the two types of fibers is also largely different, the aspect ratio of CFw being close to 2.5 and that of CF approaching 10; therefore, four times higher. The small length of CFw is a result of the mechanical treatment by ball milling for a long period of time (three cycles of 8 h each), which led to a significant reduction of both the width and length of the fibers [34].

### 3.2. Thermogravimetric Analysis (TGA)

**Figure 2:**
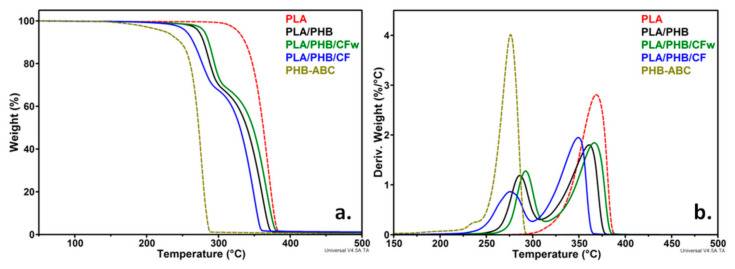
TGA (**a**) and DTG (**b**) curves of PLA, PHB-ABC, PLA/PHB, and biocomposites.

TGA and derivative curves for PLA, ABC-plasticized PHB (PHB-ABC), PLA/PHB blend, and biocomposites (PLA/PHB/CFw, PLA/PHB/CF) are shown in Figure 2. The temperature at 5% weight loss (*T_5%_*), along with the temperature of the maximum decomposition rate of PHB (*T_d-PHB_*) and PLA (*T_d-PLA_*), are presented in Table 2. 

The maximum degradation temperature of PLA is higher by about 100 °C as compared to that of plasticized PHB. This is due to both the lower *T_d_* of neat PHB and to the presence of the plasticizer, which begins to vaporize at a lower temperature. Thus, PHB-ABC showed a small shoulder at about 237 °C in the DTG diagram, which is a result of ABC vaporization [2,20]. However, this small shoulder was not visible in the blend and biocomposites, probably due to the small concentration of ABC and its dispersion in both PLA and PHB. Compared to the PLA/PHB blend, the biocomposites with CFw showed a better thermal stability, consisting in an increase in *T_5%_*, *T_d-PHB_*, and *T_d-PLA_* with 6–8 °C. On the contrary, the biocomposite with CF showed a lower thermal stability with 11–17 °C compared to the blend. 

The thermal stability of the polyesters matrix should be improved by the addition of cellulose fibers due to their high crystallinity, along with possible interactions between fibers and polyesters, which reduce the mobility of polymer chains and increase the stiffness [2,35,36]. An improved thermal stability was observed in PHB composites with cellulose fibers from wood waste due to the presence of lignin on the surface of the fibers [26]. Moreover, the thermal stability of PLA composites with cellulose fibers was improved with the gradually increase in lignin concentration in the fibers [27]. Therefore, the better thermal stability of the PLA/PHB/CFw biocomposite may be determined by the presence of lignin that acts as a thermal barrier. In addition, the antioxidant properties of lignin could also enhance the thermal stability of the PLA/PHB/CFw biocomposite [37]. The lower thermal stability of the PLA/PHB/CF biocomposite is probably due to the residual sulfate groups on the surface of the CF fibers [10,35]. The residue at 700 °C (*R_700_*) was slightly higher for the biocomposites compared to the blend and did not reflect the amount of CF or CFw in the biocomposites, due to their small concentration.

### 3.3. Differential Scanning Calorimetry (DSC)

**Figure 3:**
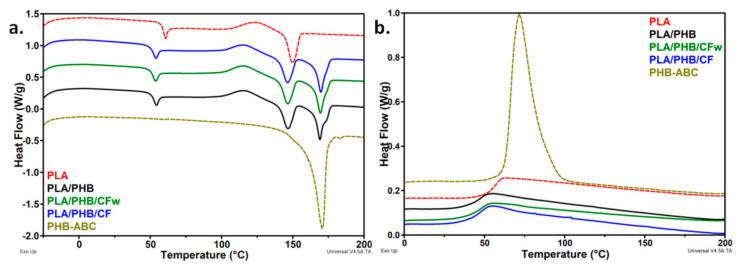
(**a**) DSC thermograms-heating cycle and (**b**) DSC thermograms-cooling cycle for the polymers, blend, and biocomposites.

The heating and cooling thermograms for PLA, plasticized PHB, the blend, and the biocomposites are shown in Figure 3. PLA exhibited a glass transition near 60 °C (Table 3), accompanied by a small endotherm caused by the relaxation of the polymer chains [9]. After the addition of plasticized PHB, the glass transition temperature (*T_gPLA_*) decreased to 52–53 °C, regardless of the presence and type of cellulose fibers. Similarly, the endothermic peak superimposed over the glass transition was shifted to a lower temperature. The decrease in *T_gPLA_* shows an increased mobility of PLA chains, probably due to the ABC plasticizer from the PHB-ABC modifier. This highlights a good dispersion of the PHB-ABC and masterbatches in PLA. The glass transition of PHB, usually around 0 °C, was not detected at this heating-cooling rate. 

Several events follow one another in the DSC thermograms of the blend and biocomposites: cold crystallization, characterized by the cold crystallization temperature (*T_ccPLA_*)/enthalpy (Δ*H_ccPLA_*), melting of PLA, characterized by the melting temperature (*T_mPLA_*)/enthalpy (Δ*H_mPLA_*), and PHB melting (Figure 3a). The *T_ccPLA_* was shifted to a lower temperature after the PHB-ABC addition in the blend and biocomposites. The greatest shift was of about 8 °C (Table 3). This may be due to the plasticizing effect of ABC that leads to an increased mobility of the PLA chains and a higher ability to crystallize [7,21]. The slight decrease in the melting temperature of PLA in the blend and the biocomposites supports an increased affinity of ABC with PLA. A similar behavior was reported for the plasticized PLA/PHB blends [38].

The crystallinity of PLA was very small in all the samples, similar to other reports [39,40]; however, that of PHB was high, but slightly decreased in the biocomposites. Comparative results of crystallinity in neat PLA and the biocomposites with cellulose fibers are given in the Supplementary Table S1. The crystallization cycle (Figure 3b) does not provide additional information: (i) a decrease in *T_gPLA_* was observed in the blend and biocomposites and (ii) only PHB crystallized at this cooling rate during DSC analysis. It is interesting that the crystallinity of PLA did not increase in the biocomposites, since cellulose reinforcements generally act as nucleating agents, increasing PLA crystallinity [2,7,21]. On the contrary, a slight decrease was observed in the case of PLA/PHB/CFw (Table 3). A decrease in PLA/PHB (75/25) crystallinity after the addition of 15 wt% ABC and, further, 5 wt% CNC, was reported in the case of electrospun fibers [24]. In this particular case, the decrease in crystallinity was explained by the increased chains’ mobility induced by the plasticizer. However, the PLA/PHB/CFw biocomposite contains a low amount of plasticizer. Comparing the crystallinity of the two biocomposites, each containing CFw and CF and the same amount of plasticizer, it may be expected that the different degree of interactions in these biocomposites could also lead to a different crystallinity. Stronger interactions between the phases may be assumed in PLA/PHB/CFw due to the presence of lignin, hindering crystallization. This was reported in the case of the PLA/CNC nanocomposites [10]. Therefore, a thorough investigation of the crystallization behavior of the PLA, PLA/PHB blend, and the biocomposites was undertaken by high-temperature XRD.

### 3.4. Temperature Dependent XRD Analysis

**Figure 4:**
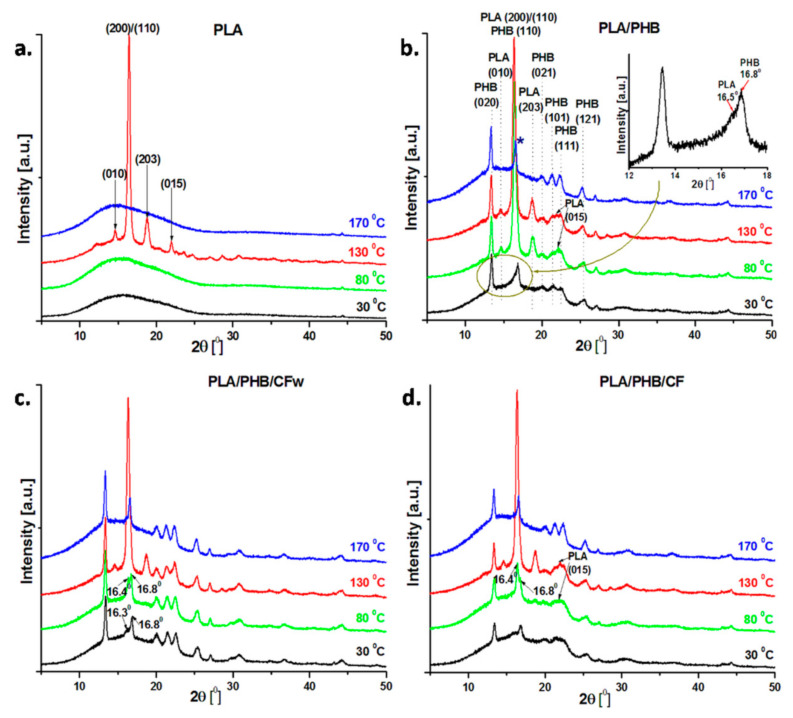
XRD diffractograms of PLA, PLA/PHB, and the biocomposites recorded at 30, 80, 130, and 170 °C during in situ high-temperature XRD analysis.

The dependence of the crystalline structure of the PLA, PLA/PHB blend and the biocomposites on temperature was analyzed by in situ high-temperature XRD. The samples were heated from room temperature to 170 °C with 10 °C/min, and the diffractograms at 30, 80, 130, and 170 °C are shown in Figure 4. These temperatures were selected based on the events observed in the DSC thermograms. The PLA was amorphous at 30 and 80 °C, but it crystallized at 130 °C (Figure 4a). The characteristic peaks of the α-form of PLA were observed at 2θ = 14.7, 16.5, 18.8, and 22.0°, corresponding to the lattice planes (010), (200)/(110), (203), and (015) [10,41].

The addition of PHB and plasticizer in PLA resulted in several changes in the crystalline structure, depending on the temperature (Figure 4b). In contrast to neat PLA, the PLA/PHB blend shows crystalline patterns starting from room temperature, with the most important peaks at 2θ = 13.4, 16.8, 20.0, 21.5, 22.6, and 25.4°, corresponding to the lattice planes (020), (110), (021), (101), (111), and (121) of PHB [42,43]. The peak at 2θ = 16.8° has a shoulder at 16.5° (see inset in Figure 4) that corresponds to the (200) or (110) crystal plan of PLA. More peaks were observed in the diffraction patterns of the blend at 80 °C (Figure 4b). Thus, new peaks appeared at 2θ = 14.7, 18.8, and 22.0°, while a high intensity peak was observed at 16.5° due to PLA crystallization. These results show that plasticized PHB underwent crystallization during storage [44], and its addition in the blend enhanced the crystallization of PLA. Indeed, the secondary crystallization in PHB during aging is well documented [45,46]. Moreover, it has been previously reported that PHB acts as a nucleation agent for PLA in PLA/PHB (75:25) blends, increasing PLA crystallinity [38]. No important changes were observed at 130 °C in the crystal structure of the blend. At a temperature above the melting temperature of PLA (170 °C), the diffraction peaks of crystalline PLA at 14.7, 16.5, 18.8, and 22.0° disappeared, while the peaks ascribed to PHB underwent minor changes (Figure 4b). At this temperature, PLA is completely fused and PHB is at the beginning of the melting process, according to DSC thermograms (Figure 3). However, it should be noted that the peak around 16.8°, corresponding to the (110) crystal plan in PHB, appeared at 2θ = 16.6° (marked with a blue star), therefore shifting to a lower 2θ diffraction angle close to the characteristic reflection peak of PLA (2θ = 16.5°). Overlapping of the PHB and PLA peaks, or larger interplanar spacing in PHB, associated with an increased degree of disorder due to the influence of PLA, may both be presumed. This may result from the good interaction between PLA and PHB, which has been already reported at this ratio in the blends [38].

Several differences were noticed in the XRD profiles of the PLA/PHB/CFw biocomposite as function of temperature when compared to the blend (Figure 4c). All the diffraction peaks of PHB appeared in the diffractograms recorded at 30 °C. In addition, a shoulder was observed at 2θ =16.3°, which comes from the main diffraction peak of PLA. At 80 °C, both peaks at 16.8° and 16.4° were enhanced, but the intensity of the peak characteristic of PLA was higher. In contrast to the blend, the crystallization of PLA in the PLA/PHB/CFw biocomposite was delayed, and it was completed only at a higher temperature. This suggests increased interactions in the biocomposites compared to the blend due to the CFw, which enhanced the interfacial adhesion and compatibility between PLA and PHB [2,40]. The peaks characteristic to the crystal structure of PLA appeared at 2θ = 14.7, 16.5, 18.8, and 22.0° in the diffractogram at 130 °C and disappeared at 170 °C (Figure 4c). Again, the peak corresponding to the (110) crystal plan in PHB appeared at 2θ = 16.6°, similar to the blend, due to the influence of PLA. 

The diffraction profiles of PLA/PHB/CF as function of temperature were similar to that those of PLA/PHB/CFw, the completely crystallized PLA being observed only at 130 °C (Figure 4d). Therefore, CF improved the interfacial adhesion between PLA and PHB and delayed the PLA crystallization [2,40]. However, several changes were noticed. Although PHB was crystallized at room temperature, the crystal peaks of PHB showed smaller intensity, and some of them collapsed. Thus, the crystallization of PHB was hindered in the biocomposites with CF, similar to other observations [47], but in contrast to the behavior of the biocomposite containing CFw. The different effect of CF compared to CFw was also observed from the diffractogram recorded at 80 °C, when the PLA peak at 16.4° was more intense than that at 16.8°, as assigned to PHB (Figure 4d). In addition, the peak of PLA at 2θ = 22.0° was quite pronounced (Figure 4d). This is in good agreement with the DSC results, where a slight increase in *X_cPLA_* was noticed. All the peaks of PLA and PHB were visible at 130 °C and only that of PHB at 170 °C.

The analysis of XRD diffractograms as functions of temperature showed several differences between the behaviors of PLA/PHB/CFw and PLA/PHB/CF. The results of DSC and in situ XRD analysis from room to melting temperature support the interactions of both CFw and CF with PLA and PHB, but suggest a different intensity of these interactions. More pronounced CFw–PLA interactions are supported by the disappearance or lower intensity of XRD peaks of crystalline PLA and the lower *X_cPLA_* value obtained by DSC. More important interactions of CF with PHB may be supposed by the lower intensity of the peaks characteristic to crystalline PHB and the lower DSC crystallinity of PHB. The difference between these biocomposites consists of the different cellulose reinforcements, for example, CFw exhibits a more hydrophobic surface due to the presence of lignin, along with a double thickness, while CF shows a hydrophilic surface due to the presence of OH groups and possible residual SO_3_H groups, along with a smaller thickness. 

### 3.5. Morphological Investigation by POM

**Figure 5:**
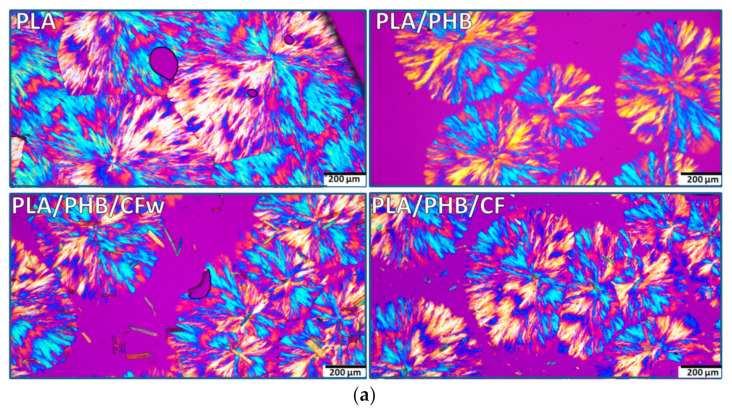

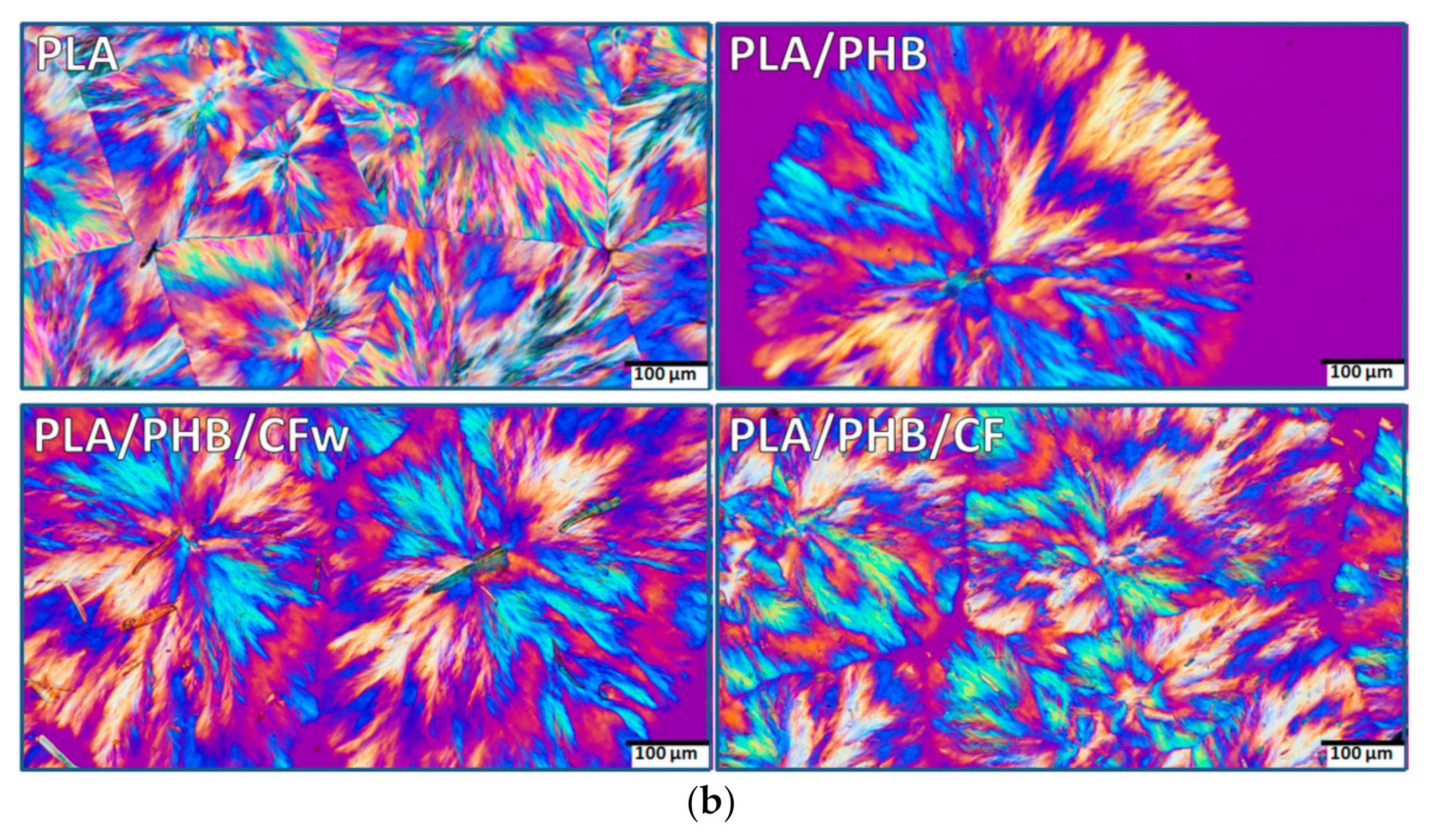
Polarized optical micrographs (different magnification) of isothermally crystallized PLA, PLA/PHB, and biocomposites at 130 °C for 24 h: (**a**) ×10; (**b**) ×20.

The spherulitic morphology has an important impact on the thermal and mechanical properties of PLA and PHB, and it can be considered as a key element in the discussion of their properties. Therefore, the samples were isothermally crystallized for 24 h at 130 °C, the temperature at which PLA was completely crystallized according to XRD data, and analyzed by POM (Figure 5).

The POM images of PLA show a complex morphology at both magnifications, with impinged spherulites and straight boundaries (Figure 5a,b—PLA). The spherulites consist of fibrillar crystalline structures grown in radial directions from a central nucleus, forming alternate blue/orange birefringence stripes. Extinction bands are also visible, but they are distorted and irregular so that the structures cannot be identified as ring banded spherulites. It can be presumed that the crystalline lamellae did not twist cooperatively to form banded spherulites [48]. The size of the spherulites ranges between 500 and 800 µm, with an average size around 600 µm. 

In the PLA/PHB blend, the alternate optical blue/orange birefringence stripes are better defined and the crystalline structure appears to be more regular (Figure 5a,b–PLA/PHB). In addition, the size of the spherulites is reduced to 400–600 μm. This is an effect of both PHB, acting as a nucleating agent, and of the ABC plasticizer [21]. The addition of cellulose fibers, CFw or CF, led to a further decrease in spherulites’ size to 300–500 μm, with an average dimension around 400 μm (Figure 5a,b—PLA/PHB/CFw and PLA/PHB/CF). This confirms their nucleating effect. Impinged spherulites were frequently observed in each biocomposite. It should be noted that micron-sized fibers were frequently noticed in the PLA/PHB/CFw biocomposite, while only rarely seen and difficult to distinguish in the PLA/PHB/CF. The results related to the different fibers sizes are due to the different applied treatments: mechanical treatment for the preparation of CFw and acid hydrolysis treatment in the case of CF. 

**Figure 6:**
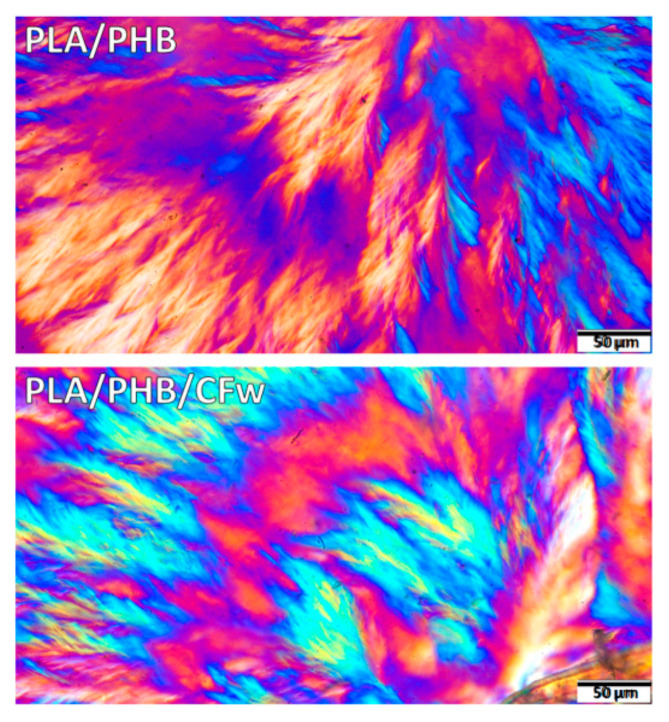
Polarized optical micrographs (×40) of isothermally crystallized PLA/PHB and PLA/PHB/CFw biocomposite at 130 °C for 24 h.

A more detailed analysis of the spherulitic structure of isothermally crystallized PLA/PHB and PLA/PHB/CFw biocomposite at 130 °C for 24 h is given in Figure 6. A fern leaf-like fibrillar architecture [49] with bended and branched fibrillar lamellae can be observed in this figure. In several cases, alternating birefringence colors were observed in the same spherulite quadrant (Figure 6): the radially oriented lamellae show one color (orange) and the branching lamellae oriented tangentially show the opposite color (blue). This is probably due to approximately perpendicular orientations of the branches [50]. These effects are more frequent in the PLA/PHB/CFw biocomposite, with orange traces being observed in the blue region. A ring-banded morphology with alternate birefringence rings, involving crystal twisting [50], was more clearly observed in the biocomposite.

### 3.6. Dynamic Mechanical Analysis

**Figure 7:**
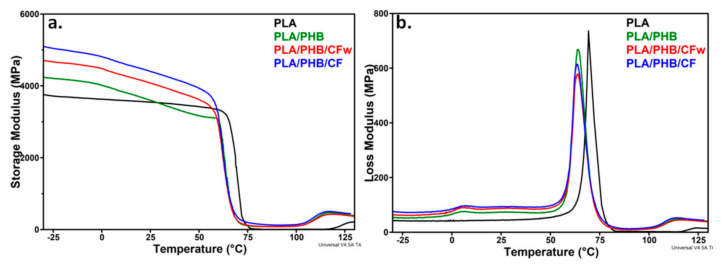
Storage modulus (**a**) and loss modulus (**b**) of PLA, PLA/PHB and biocomposites against temperature.

The original curves of the storage modulus and loss modulus vs. temperature for the PLA, PLA/PHB blend, and the biocomposites are shown in Figure 7. As a measure of the materials stiffness, the storage modulus (*E′*) of PLA showed a small variation with temperature up to the glass transition temperature (*T_gPLA_*), when a sudden decrease occurs due to the increased mobility of the PLA chains; this is followed by the rubbery plateau, when the *E′* value is small and relatively constant with temperature, and then cold crystallization at temperatures over 120 °C.

A slight increase in the *E′* was observed at temperatures bellow room temperature and over 75 °C after the addition of plasticized PHB in PLA. A strong shift of the glass transition and cold crystallization temperatures toward lower values was noticed in both *E′* and loss modulus (*E″*) curves, which is an effect of PHB and plasticizer addition [21]. A similar effect was highlighted by DSC (Table 3). The presence of CF or CFw in biocomposites has an important effect on the storage modulus that is higher than that of PLA/PHB, regardless the temperature, and also higher than that of PLA on nearly the entire temperature range (Figure 7a). Thus, the addition of CF and CFw containing masterbatches increased the *E′* of PLA by 36 and 24% at −25 °C, by 26 and 15% at 25 °C, and by 100 and 22% at 75 °C (Table 4). This shows the stiffening effect of cellulose fibers, especially of CF, similar to other observations [11,51]. Comparative results of the storage modulus at room temperature are given in the Supplementary Table S1. It may be observed that the biocomposites from this study showed similar or higher storage modulus values compared to those previously reported [52,53]. The higher storage modulus of the biocomposite containing CF compared to that containing CFw can be explained by the higher aspect ratio and shorter width of these fibers. Indeed, a longer fiber length and higher aspect ratio ensures a more efficient stress transfer from the polymer to the fibers and therefore, a higher stiffness of the composite [54]. However, CFw also improved the storage modulus, although it has a double width and a four times lower aspect ratio, probably due to the beneficial effect of lignin. 

The variation of the *E″* with temperature (Figure 7b) showed that the glass transition of PHB was not significantly influenced by the presence of CF or CFw, but *T_gPLA_* (69.3 °C) was shifted to lower values after the addition of plasticized PHB (64.1 °C), similar to the DSC results. This was due to the plasticizing effect of ABC [21]. The increased area under the *E″* peak in the case of the biocomposites compared to PLA should be noted, which indicates an increased toughness. 

### 3.7. Morphological Investigation of Biocomposites by AFM

**Figure 8:**
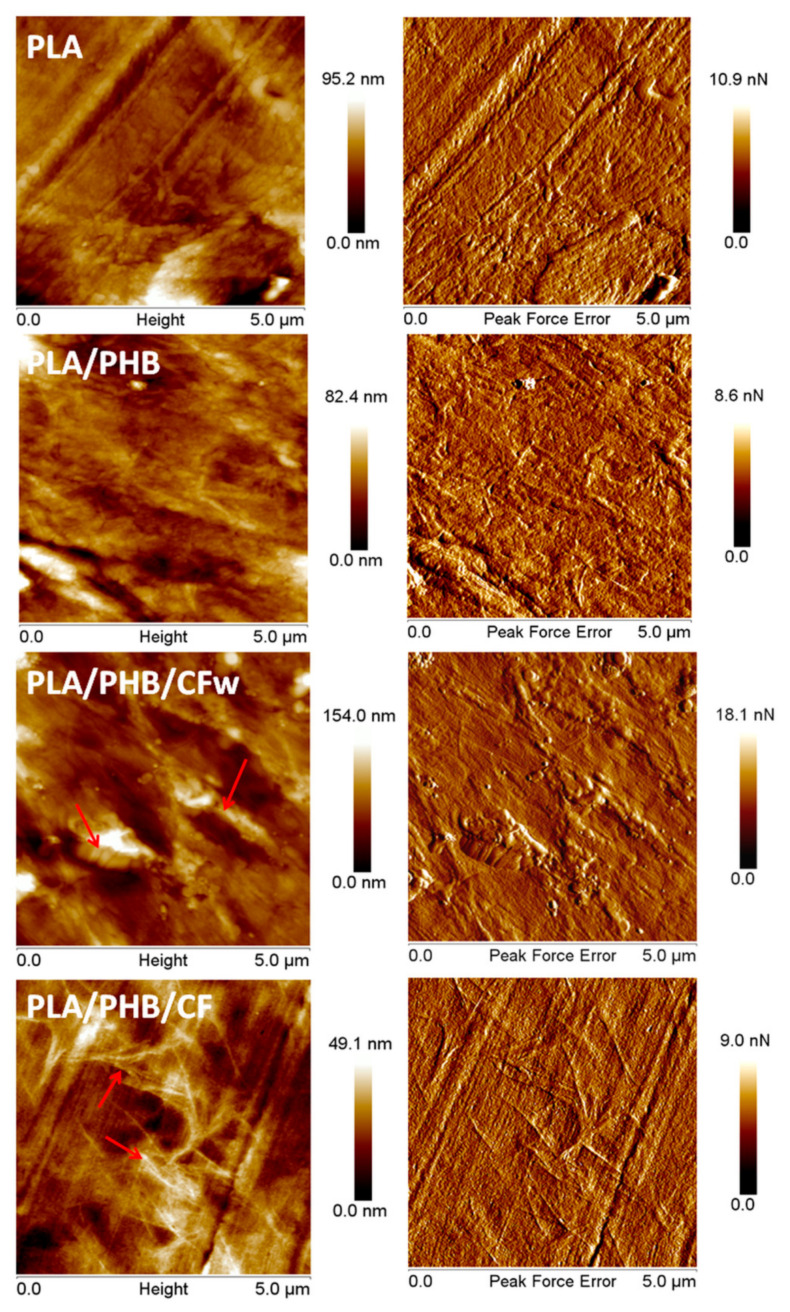
Topographic and PFE images of PLA, PLA/PHB, and biocomposites.

PF QNM mode of AFM was used to characterize the surface morphology of the biocomposites. The AFM images of PLA (Figure 8) show a typical amorphous morphology without any crystalline organization, which was also confirmed by XRD analysis of PLA at room temperature (Figure 4a). 

Several light-colored objects, like that in the bottom right corner in Figure 8–PLA, were observed on the surface of PLA, which is a commercial product containing additives (fillers, nucleating agents, residual catalyst) [55]. The channels and stripes observed on the surface of the PLA film come from the compression molding plates. The PLA/PHB blend shows a similar surface morphology (Figure 8–PLA/PHB), except for several zones with a fibrillar morphology characteristic to the PHB component [56]. This was also highlighted by XRD (Figure 4b), which demonstrates the presence of PHB crystalline peaks even at room temperature. 

In the AFM images of PLA/PHB/CFw (Figure 8), both large and very small cellulose fibers were observed. The small cellulose fibers appear as white points and are seen well dispersed on the surface of the sample. The presence of large fibers, marked with arrows, was due to the inexpensive mechanical treatment applied to obtain CFw. However, they are well covered with polymer, which indicates a good interface, probably an effect of the lignin present on their surface. The crystalline structures of PHB were barely detected on the surface of PLA/PHB/CFw, due to the large size of several CFw. 

A completely different surface morphology was noticed in the case of PLA/PHB/CF (Figure 8). Interconnected networks of fibrils were observed, as marked with arrows. These may come from the crystalline structures of PHB or from the cellulose fibers. XRD patterns showed a lower crystallization tendency in PLA/PHB/CF at room temperature, compared to the other samples, and DSC showed a lower crystalline degree in this case; therefore, the fibrils network observed in the AFM images is likely composed of CF-type cellulose fibers. The good dispersion of large aspect ratio CF observed by AFM may also explain the good mechanical properties highlighted by DMA in the case of PLA/PHB/CF.

## 4. Conclusions

The effect of wood-waste-derived cellulose fibers containing lignin compared to those obtained from pure cellulose on the properties of a plasticized PLA/PHB blend was studied for the first time. PLA was modified with plasticized PHB and cellulose fibers using a melt compounding masterbatch technique for the better dispersion of additives. The thermal stability of the biocomposites was improved by the cellulose fibers obtained from wood waste, CFw, using mechanical treatment, while the dynamic mechanical properties noticeably increased in the presence of cellulose fibers obtained from pure cellulose, CF, using acid hydrolysis. DSC and in situ XRD results suggest preferential interactions of CFw with PLA and CF with PHB. The CFw–PLA interactions were supported by the disappearance or lower intensity of XRD peaks of crystalline PLA and the lower *X_cPLA_* value obtained by DSC, while the interactions of CF with PHB were supported by the lower intensity of the peaks characteristic to crystalline PHB and the slightly lower DSC crystallinity of PHB in this biocomposite. According to AFM analysis, the distinct size and aspect ratio, together with the different surface properties of the two cellulose fibers, were responsible for the dissimilar influence on PLA’s overall properties. The use of cheap cellulose fibers from wood waste may lead to PLA/PHB biocomposites with good thermal and mechanical properties, similar to those containing cellulose fibers obtained by acid hydrolysis. Together, these qualities recommend the PLA/PHB/CFw biocomposite as a promising substitute of fossil based materials in packaging applications. In addition, in situ XRD analysis over a large temperature range has proven to be a useful technique to better understand changes in the crystal structure of complex biomaterials.

## Figures and Tables

**Table 1:** Composition of the samples.

Sample	PLA	PHB	ABC	CFw	CF
PLA	100	-	-	-	-
PHB-ABC	-	90	10	-	-
PLA/PHB	71.1	26.0	2.9	-	-
PLA/PHB/CFw	69.9	25.7	2.9	1.5	-
PLA/PHB/CF	69.9	25.7	2.9	-	1.5

**Table 2:** Thermal degradation characteristics of PLA/PHB biocomposites.

Biocomposites	*T_5%_* [°C]	*T_d-PHB_* [°C]	*T_d-PLA_* [°C]	*R_700_* [%]
PLA	327.5	-	368.7	0.79
PHB-ABC	225.6	276.0	-	0.23
PLA/PHB	272.6	286.0	361.3	0.69
PLA/PHB/CFw	280.4	292.2	366.8	0.93
PLA/PHB/CF	255.4	275.0	349.6	1.04

**Table 3:** DSC data for the PLA, PHB, PLA/PHB blend, and biocomposites.

Biocomposites	*T_gPLA_* [°C]	*T_ccPLA_* [°C]	Δ*H_ccPLA_* [J/g]	*T_mPLA_* [°C]	Δ*H_mPLA_* [J/g]	T_mPHB_ * [°C]	ΔH_mPHB_ * [J/g]	*T_c_* [°C]	Δ*H_cPHB_* [J/g]	*X_cPLA_* [%]	*X_cPHB_* [%]
PLA	59.7	124.5	14.3	149.8	20.7	-	-	-	-	6.9	-
PHB-ABC	-	-	-	-	-	170.6	94.5	71.8	76.0	-	71.9
PLA/PHB	52.8	116.8	16.8	146.6	21.4	169.0	26.6	-	-	7.0	69.3
PLA/PHB/CFw	52.2	116.4	17.0	146.5	20.7	169.4	25.5	-	-	5.7	68.5
PLA/PHB/CF	52.3	116.4	15.7	146.4	20.6	169.7	24.8	-	-	7.5	66.7

* *T_mPHB_* and Δ*H_m PHB_* are the melting temperature and enthalpy of PHB.

**Table 4:** Characteristics of PLA and PLA/PHB biocomposites from DMA curves.

Biocomposites	*E^′^_-25_* [MPa]	*E^′^_25_* [MPa]	*E^′^_75_* [MPa]	*T_gPHB_* [°C]	*T_gPLA_* [°C]	*T_ccPLA_* [°C]
PLA	3716	3548	98.8	-	69.3	124.1
PLA/PHB	4210	3599	107.6	6.9	64.1	115.7
PLA/PHB/CFw	4667	4079	120.1	5.1	63.9	115.4
PLA/PHB/CF	5043	4404	197.1	7.4	63.5	114.0

## Data Availability

Data supporting the reported results are available on request from the corresponding author.

## Supplementary Materials

The following supporting information can be downloaded at: https://www.mdpi.com/article/10.3390/molecules27082390/s1, Table S1: Comparative results of crystallinity and storage modulus for neat PLA and PLA biocomposites from the literature and from this work.

## Sample Availability

Samples of the compounds are available from the authors.
