# Supplementary material for: Poly(lactic acid)/Poly(3-hydroxybutyrate) Biocomposites with Differently Treated Cellulose Fibers
Source: Molecules. 2022 Apr 7;27(8):2390. doi: 10.3390/molecules27082390 (PMC9032581; doi:10.3390/molecules27082390)
Supplement: Supplementary file 1 [file molecules-27-02390-s001.zip › molecules-1649093-supplementary.pdf]

Supplementary Materials

Poly(lactic Acid)/poly(3-hydroxybutyrate) Biocomposites with Differently Treated Cellulose Fibers

Adriana Nicoleta Frone, Marius Ghiurea, Cristian Andi Nicolae, Augusta Raluca Gabor, Stefania Badila and Denis Mihaela Panaitescu *

National Institute for Research & Development in Chemistry and Petrochemistry—ICECHIM, 202 Splaiul Independentei, 060021 Bucharest, Romania; adriana.frone@icechim.ro (A.N.F.); ghiurea@gmail.com (M.G.); cristian.nicolae@icechim-pd.ro (C.A.N.); raluca.gabor@icechim.ro (A.R.G.); stefania.badila@yahoo.com (S.B.)

* Correspondence: panaitescu@icechim.ro;

Table S1. Comparative results of crystallinity and storage modulus for neat PLA and PLA biocomposites from the literature and from this work.

Biocomposites	Fiber mass content (%)	Storage modulus (room temperature) (MPa)	PLA crystallinity (%)*	References
Neat PLA	-	-	1.3	[39]
Neat PLA	-	-	5.6	[40]
Neat PLA	-	3400	-	[52]
Neat PLA	-	3548	6.9	This study
PLA/CNC	5	-	2.0	[39]
PLA/CF	15	4329	-	[53]
PHB/PLA/CF	4	2900	-	[52]
PLA/PHB/CFw	5	4079	5.7	This study
PLA/PHB/CF	5	4404	7.5	This study

*Determined from DSC analysis

References

[39] Vilarinho, F.; Stanzione, M.; Buonocore, G.G.; Barbosa-Pereira, L.; Sendón, R.; Vaz, M.F.; Silva, S.A. Green tea extract and nanocellulose embedded into polylactic acid film: Properties and efficiency on retarding the lipid oxidation of a model fatty food. *Food Packag. Shelf Life* **2021,** *27*, 100609.

[40] Arrieta, M.P.; Fortunati, E.; Dominici, F.; Rayón, E.; López, J.; Kenny, J.M. Multifunctional PLA–PHB/cellulose nanocrystal films: Processing, structural and thermal properties. *Carbohydr. Polym.* **2014**, *107*, 16-24.

[52] Aydemir, D.; Gardner, D.J. Biopolymer blends of polyhydroxybutyrate and polylactic acid reinforced with cellulose nanofibrils. *Carbohydr. Polym.* **2020**, *250*, 116867.

[53] Singh, A.A.; Genovese, M.E.; Mancini, G.; Marini, L.; Athanassiou, A. Green Processing Route for Polylactic Acid–Cellulose Fiber Biocomposites. *ACS Sustain. Chem. Eng.* **2020**, *8*, 4128-4136.
